# Type 1 fimbriae-mediated collective protection against type 6 secretion system attacks

**DOI:** 10.1128/mbio.02553-23

**Published:** 2024-03-18

**Authors:** Margot Marie Dessartine, Artemis Kosta, Thierry Doan, Éric Cascales, Jean-Philippe Côté

**Affiliations:** 1 Département de biologie, Faculté des sciences, Université de Sherbrooke, Sherbrooke, Quebec, Canada; 2 Plateforme de microscopie, Institut de Microbiologie de la Méditerranée (IMM, FR3479), Aix-Marseille Univ, CNRS, Marseille, France; 3 Laboratoire d'Ingénierie des Systèmes Macromoléculaires (LISM, UMR7255), Institut de Microbiologie de la Méditerranée, Aix Marseille Univ, CNRS, Marseille, France; University of Michigan-Ann Arbor, Ann Arbor, Michigan, USA

**Keywords:** bacterial competition, type 6 secretion system, Keio collection, microcolony formation, type 1 fimbriae

## Abstract

Bacterial competition may rely on secretion systems such as the type 6 secretion system (T6SS), which punctures and releases toxic molecules into neighboring cells. To subsist, bacterial targets must counteract the threats posed by T6SS-positive competitors. In this study, we used a comprehensive genome-wide high-throughput screening approach to investigate the dynamics of interbacterial competition. Our primary goal was to identify deletion mutants within the well-characterized *E. coli* K-12 single-gene deletion library, the Keio collection, that demonstrated resistance to T6SS-mediated killing by the enteropathogenic bacterium *Cronobacter malonaticus*. We identified 49 potential mutants conferring resistance to T6SS and focused our interest on a deletion mutant (∆*fimE*) exhibiting enhanced expression of type 1 fimbriae. We demonstrated that the presence of type 1 fimbriae leads to the formation of microcolonies and thus protects against T6SS-mediated assaults. Collectively, our study demonstrated that adhesive structures such as type 1 fimbriae confer collective protective behavior against T6SS attacks.

**IMPORTANCE:**

Type 6 secretion systems (T6SS) are molecular weapons employed by gram-negative bacteria to eliminate neighboring microbes. T6SS plays a pivotal role as a virulence factor, enabling pathogenic gram-negative bacteria to compete with the established communities to colonize hosts and induce infections. Gaining a deeper understanding of bacterial interactions will allow the development of strategies to control the action of systems such as the T6SS that can manipulate bacterial communities. In this context, we demonstrate that bacteria targeted by T6SS attacks from the enteric pathogen *Cronobacter malonaticus*, which poses a significant threat to infants, can develop a collective protective mechanism centered on the production of type I fimbriae. These adhesive structures promote the aggregation of bacterial preys and the formation of microcolonies, which protect the cells from T6SS attacks.

## INTRODUCTION

Bacteria are often found within complex microbial communities where constant interactions occur. Effective competition strategies are essential for survival, especially in environments where nutrients and micro-elements (e.g., iron) are limited (1). For instance, some microorganisms secrete antimicrobial molecules targeting surrounding microbes (2). In bacteria, competitive interactions are often mediated by secretion systems (3, 4). Secretion systems are protein complexes that allow the passage of proteins across the cell envelope and are involved in bacterial virulence by promoting the invasion of eukaryotic cells or scavenging resources in the environment (5, 6). Twelve secretion systems have been identified in bacteria. Among them, the type 6 secretion system (T6SS) is a potent weapon that delivers toxins into surrounding bacterial cells (7, 8). The T6SS also contributes to virulence by driving the remodeling of microbial communities by pathogenic bacteria and facilitating the establishment of T6SS-positive pathogens (9–11).

The T6SS is a harpoon-like contractile nanomachine that triggers interbacterial competition through the injection of toxic effectors directly into target cells in a contact-dependent manner (12, 13). This nano-harpoon is composed of a membrane complex and a baseplate that serves as a docking station for the assembly of a tail tube/sheath complex (14–16). The tail tube is composed of stacked Hcp hexamers wrapped by a sheath formed by the TssB and TssC proteins (17, 18). The tube is capped by the spike, comprising the VgrG trimer sharpened with the PAAR-domain protein (19). Effector proteins displaying diverse functions [phospholipases (20), amidases (21), DNases (22), etc.] (23) can be loaded within the lumen of the Hcp tube or linked as cargo to VgrG/PAAR domain proteins directly or with the support of specific adaptors (12, 24, 25). The sheath is polymerized in an extended conformation, and upon its contraction, the tail tube is pushed across the envelope of both attacker and target cells, so the antibacterial T6SS effectors can be delivered directly into target cells to kill them.

Target cells can display defense mechanisms against T6SS attacks. The main defense system is orchestrated by immunity proteins that usually specifically bind to the effectors (13). This approach is also used by attacker cells to avoid autointoxication. Immunity-independent resistance to T6SS has also been reported. Indeed, *Escherichia coli* can sense T6SS effectors through envelope stress-response pathways and resists T6SS attacks from *Vibrio cholerae* (26). Moreover, target cells can modulate environmental parameters influencing the interaction between attacker and target cells. For example, introducing spatial separation between target and attacker cells is an effective mechanism of resistance to contact-dependent attacks by T6SS (27, 28). This spatial segregation may be due to the production of exopolysaccharides (EPS) to form a physical barrier (29) or biofilm formation (30) which can lead to the formation of microcolonies protecting cells located at the center, by the formation of a barrier of dead cells and debris at the attacker-target interface (28, 31). Collective behaviors are widespread in microbial environment, such as the intestinal microbiota (32, 33), where T6SS performs crucial functions to modulate population dynamics between host-associated microbes, enteric pathogens, and host immune response (34, 35). Despite growing interest in resistance mechanisms, many aspects remain unknown regarding target cell defenses against T6SS assaults.

High-throughput approaches using ordered libraries of mutants allow to rapidly discover and investigate new genetic pathways involved in a phenotype of interest (36, 37), including the regulation of T6SS (38). In this study, we developed a screening method that enabled us to analyze interbacterial competition in a genome-wide high-throughput manner. Using *Cronobacter malonaticus,* an *Enterobacteria* highly prevalent in powdered infant formula and known to cause severe infections in newborns and immunocompromised individuals (e.g., meningitis, necrotizing enterocolitis) (39), as a model attacker bacterium*,* and the single-gene deletion mutants for all nonessential *E. coli* K-12 genes library (Keio collection) (40) as target cells, we identified that the deletion of the *fimE* recombinase provides resistance to T6SS attacks. We further showed that a Δ*fimE* mutant overexpressed type 1 fimbriae leading to the formation of microcolonies. Target cells within these microcolonies were protected from contact-dependent killing mediated by the T6SS, leading to their survival. This defense mechanism could represent a nonspecific resistance mechanism, unlike immunity proteins, which could potentially lead to resistance against a broad range of assailants.

## RESULTS

### *Cronobacter malonaticus* 3267 efficiently kills *E. coli* in a T6SS-dependent manner

**Fig 1:**
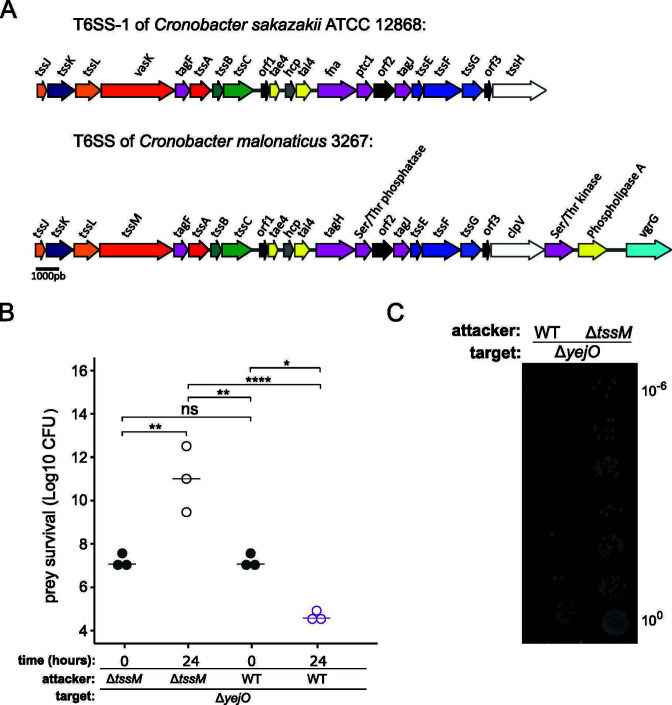
*C. malonaticus* 3267 kills *E. coli* BW25113 in a T6SS-dependent manner. (**A**) Schematic representation of the T6SS-1 gene cluster in *C. sakazakii* ATC12868 (from Wang et al. [41]) and of the T6SS gene cluster in *C. malonaticus* 3267. Structural genes of the membrane complex are displayed in light to dark orange. Genes coding for baseplate proteins are displayed in light to dark blue. The genes *tssB* and *tssC* of the tail tube/sheath complex are shown in light and dark green. Genes coding for proteins involved in T6SS post translational regulation are indicated in pink. Genes coding for toxins and antitoxins are displayed in yellow. Open reading frame with unknown function is shown in black. (**B**) Attackers and kanamycin-resistant target cells were mixed at a concentration of 10^6^ CFU and at a ratio of 1:1 and incubated onto a nitrocellulose filter for 24 h. Surviving target cells at 0 and 24 h were enumerated by plating on selective agar plates. Target survival is presented in log10 CFUs. (**C**) Representative picture of the target cells selection after the killing assay. One-way ANOVA test was performed to determine statistical significance (ns = nonsignificant, *P ≤ 0.05, **P ≤ 0.005, ****P ≤ 0.0001). Data represent three independent experiments.

A recent study has shown that a conserved T6SS gene cluster among *Cronobacter sakazakii* strains displays a strong activity in laboratory conditions (41). Based on this study, we hypothesized that *Cronobacter malonaticus* strain 3267 may also possess a functional T6SS. Sequencing confirmed the presence of a T6SS gene cluster in *C. malonaticus* 3267, bearing similarities to the T6SS-1 found in *C. sakazakii* ATCC 12868 (Fig. 1A). A bacterial competition assay validated that *C. malonaticus* 3267 displays antibacterial activity against *E. coli* in typical laboratory growth conditions (Fig. 1B). We used *E. coli* BW25113 Δ*yejO* from the Keio collection as target cells, which we consider as wild-type (WT) *E. coli* in our study. *yejO* is a pseudogene in *E. coli*, inactivated by an insertion sequence (IS5K) close to its start codon. BW25113 Δ*yejO* target is eliminated to a similar extent than *E. coli* BW25113 WT strain, thus making it a suitable target cell in our study (Fig. S1A).

To test whether the antibacterial activity of *C. malonaticus* was T6SS dependent, we quantified the survival of *E. coli* Δ*yejO* target cells after co-incubation with a WT or a T6SS-inactive mutant of *C. malonaticus* 3267 (Δ*tssM*). When *E. coli* Δ*yejO* is incubated with WT *C. malonaticus* 3267, ~5 × 10^4^ CFUs of target cells were recovered after the competition assay (Fig. 1B and C). However, when co-incubated with the Δ*tssM* mutant, ~1 × 10^12^ CFUs of target cells were recovered. The deletion of *tssM* did not affect the growth of *C. malonaticus* 3267 (Fig. S1B), and the presence of target cells did not affect *C. malonaticus* 3267 (Fig. S1C). Collectively, these data suggest that *C. malonaticus* 3267 employs its T6SS to eliminate *E. coli* target cells.

### A high-throughput interbacterial competition assay identifies *E. coli* Δ*fimE as* resistant to T6SS

**Fig 2:**
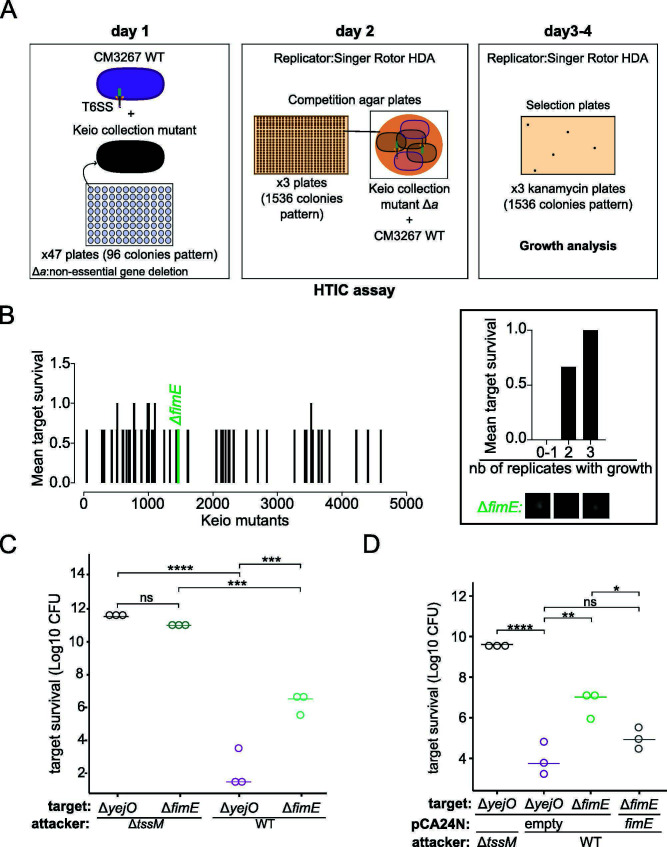
High-throughput interbacterial competition assay identifies *E. coli* BW25113 Δ*fimE* mutant as resistant to *C. malonaticus* 3267 T6SS-mediated killing. (**A**) Schematic representation of the HITC assay methodology (CM3267: *C. malonaticus* 3267). (**B**) Bar plot of mutant survival after the HTIC assay. For each replicate, survival was assessed using binary value; colony growth = 1, no colony growth = 0. In this setting, a mutant is resistant if there is a surviving colony in at least two of the three replicates. (**C**) Interbacterial competition assay between WT or Δ*tssM* attackers and Δ*yejO* or Δ*fimE* target cells. (**D**) Complementation of Δ*fimE* mutant with pCA24N plasmid (empty vector or *fimE*) to restore the susceptibility to T6SS. (**C and D**) Target survival is presented in log10 CFU. One-way ANOVA test was performed to determine statistical significance (ns = nonsignificant, *P ≤ 0.05, **P ≤ 0.005, ***P ≤ 0.0005, ****P ≤ 0.0001). Data represent three independent experiments.

High-throughput interbacterial competition (HTIC) assays combined with the use of target or attacker libraries of mutants are effective in uncovering new mechanisms involved in T6SS function and biogenesis (38). We performed a high-throughput competition assay between the Keio collection mutants as target cells and *C. malonaticus* 3267 as the attacker strain (Fig. 2A). We co-cultured targets and attackers on competition plates in a 1,536-colony density format. After 24 h of co-incubation, colonies were replicated on selection plates containing kanamycin, allowing the growth of the Keio mutants that escaped T6SS killing. We expected that the deletion of genes related to T6SS susceptibility would increase survival of prey cells upon interbacterial competition.

To avoid bias in determining which genes confer resistance to the T6SS attack, we used binary values, where 1 means growth and 0 means no growth, instead of measuring colony size or colony density. Keio mutants can display different colony morphologies (e.g., small, mucoid, and sticky) (42), and therefore, measuring colony size on the selection plates does not correlate with the resistance level of target cells. The binary values of three replicates were averaged in order to score the mutants in the Keio collection (Fig. 2B).

Of the ~4,000 Keio mutants, only 49 displayed resistance (Table S2) to T6SS in at least two out of the three replicates as some defense mechanisms can provide only partial resistance to T6SS (27). These resistant mutants included the *fimE* mutant. In pairwise interbacterial competition assays against the WT attacker, the *fimE* mutant displayed a difference of almost 4 log CFUs in survival compared to the *yejO* mutant (Fig. 2C). However, the *fimE* mutant still showed lower survival than target cells in competition with the Δ*tssM* attacker (~4 log CFUs), suggesting that the *fimE* mutant conferred only partial resistance to T6SS attacks (Fig. 2C). Transcomplementation using the high-copy pCA24N-*fimE* plasmid (43) restored the killing by *C. malonaticus* 3267 to WT levels (Fig. 2D), confirming that the observed phenotype is the result of the deletion of the *fimE* gene.

### *E. coli BW25113* Δ*fimE* exhibits increased production of type 1 fimbriae

The *fim* operon is composed of nine genes encoding structural, assembly, and transport components (*fimAICDFGH*) as well as regulators (*fimB* and *fimE*). FimA is the major structural subunit of the fimbriae and is secreted by the FimC chaperone and the FimD usher (44). FimH encodes a mannose-specific adhesin located at the tip of the fimbriae that mediates the attachment to mannosylated receptors at the cell surface. FimB and FimE encode recombinases that control the permutation of an invertible 314 bp element (*fim* switch) located directly in front of *fimA* and flanked by two inverted 9 bp repeats (45, 46). This inversion activates or prevents the transcription of type 1 fimbriae genes in a process called phase variation (47). The FimE recombinase preferentially switches from a phase with fimbriae production (ON-phase) to a phase without fimbriae production (OFF-phase), whereas FimB recombinase mediates permutations in both directions, with a preference to an ON-phase orientation (48, 49).

**Fig 3:**
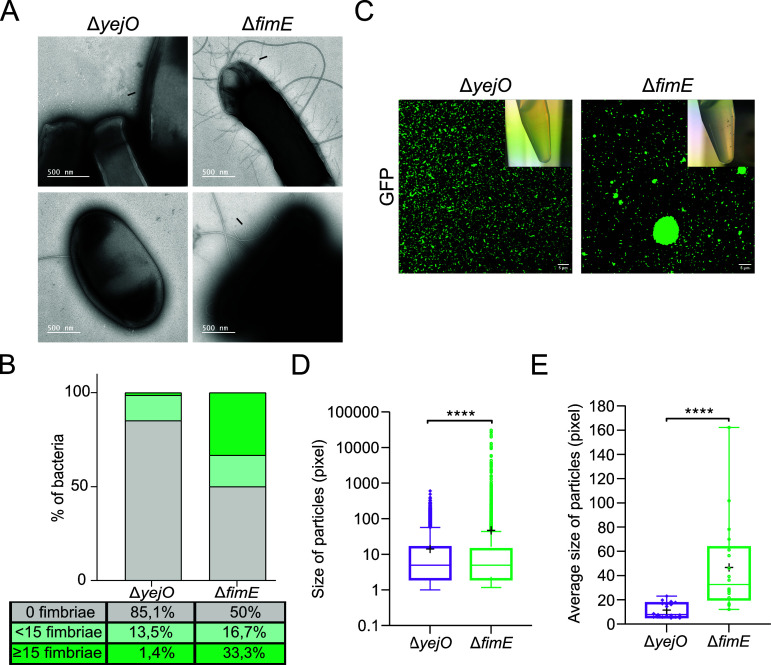
The Δ*fimE* mutant produces type 1 fimbriae that promote the formation of bacterial microcolonies. (**A**) Electron microscopy of fimbriated cells. Negatively stained preparation of a 24-h stationary culture of *E. coli* BW25113 Δ*yejO* and Δ*fimE*, grown on Luria Broth agar plate. (**B**) Proportion of fimbriated and nonfimbriated cells in Δ*yejO* (*n* = 74; 14.9% of fimbriated cells) and Δ*fimE* (*n* = 60; 50% of fimbriated cells). (**C**) gfp-Expressing strains were spotted in duplicate after centrifugation and vortex cycle and observed in fluorescence microscopy. Three images were taken at random points for each duplicate for three independent experiments. Microcolonies size was assessed using ImageJ. (**D**) Box plot of the particle sizes of the Δ*yejO* and Δ*fimE* mutants. Box plots extend from the 5th to 95th percentiles. Cross, mean; crossing line, median. Nonparametric Mann Whitney test was performed to determine statistical significance (****P < 0.0001). (**E**) Box plot of the average size of particles of each random point images (*n* = 18). Unpaired *t*-test was performed to determine statistical significance (****P < 0.0001). Box plots extend from the minimum to the maximum, showing all points. Cross, mean; crossing line, median.

The overall effect of the FimE recombinase on the expression of fimbriae is still debated. Orndorff et al. demonstrated an increase in the number of fimbriae per cell in *E. coli* when producing the *fim* operon from a high copy number plasmid containing a deletion of the *fimE* gene (50). However, another study showed no difference in bacterial fimbriation in the isogenic *fimE* mutant in *E. coli* MG1655 compared to the WT strain (51). Therefore, we first analyzed the production of type 1 fimbriae of the *E. coli* BW25113 *fimE* mutant. Electron microscopy revealed that 50% of the Δ*fimE* cells possessed fimbriae compared to only 15% of Δ*yejO* cells (Fig. 3A and B). Moreover, when Δ*fimE* cells were fimbriated, there were more fimbriae per cell, with 33% of cells having more than 15 fimbriae on their surface, compared to only 1.4% of Δ*yejO* cells (Fig. 3B).

We also observed that when centrifuged, the *fimE* mutant formed aggregates that did not dissociate easily. Shear stress is known to enhance the adhesion of bacteria via the FimH adhesin of type 1 fimbriae (52). Therefore, we next observed by microscopy if the increased production of type 1 fimbriae in the *fimE* mutant led to the formation of microcolonies (Fig. 3C). Bacterial cultures were centrifuged and vortexed, then spotted onto semi-solid Luria broth (LB) agar, and observed on a confocal microscope. Only the *fimE* deletion mutant was able to form large microcolonies. The area of all particles was analyzed to assess the size of microcolonies (Fig. 3D). Δ*fimE* strain had a significantly greater average particle size compared to the Δ*yejO* strain (Fig. 3E). The observed variability within the size of microcolonies for Δ*fimE* strains correlates with the electron microscopy findings, showing that 50% of cells produce fimbriae. Overall, these results show that the *fimE* deletion mutant actively produces type 1 fimbriae and forms microcolonies when subjected to shear forces.

### Type 1 fimbriae mediate the formation of microcolonies

We next investigated whether the formation of microcolonies was linked to the FimH adhesin located at the tip of the type 1 fimbrial structure. Our goal was to determine whether the microcolonies were responsible for the resistance to T6SS attacks or whether the fimbriae themselves were sufficient to obstruct T6SS assaults. Because of their length (~1 µm) and their considerable number on the cell surface (>200/cell) (44, 53), type 1 fimbriae could mask direct interactions between cell structures and the environment. One possibility is thus that fimbriated cells are shielded against T6SS by the fimbriae covering their surface.

**Fig 4:**
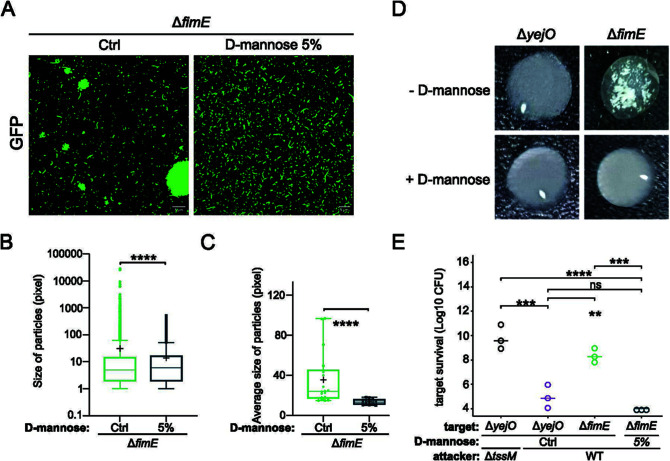
Type 1 fimbriae adhesin is essential for microcolony formation and T6SS resistance. (**A**) Visualization of microcolonies using fluorescence microscopy. (**B**) Yeast agglutination assay with and without D-mannose (competitive ligand binding to FimH). Agglutination phenotype is observed for the Δ*fimE* mutant. The absence of agglutination is characterized by a cloudy appearance of the mixture. (**C**) Box plot of the overall size of particles distribution of the Δ*fimE* mutant with and without 5% of D-mannose. Box plots extend from the minimum to the maximum, showing all points. Cross, mean; crossing line, median. Nonparametric Mann Whitney tests were performed to assess statistical significance using R (****P ≤ 0.0001). (**D**) Box plot of the average size of particles of each random point images (*n* = 18). Box plots extend from the 5th to 95th percentiles. Cross, mean; crossing line, median. Unpaired *t*-test was performed to assess statistical significance using R (**P = 0.005). (**E**) Interbacterial competition assay between WT or Δ*tssM* attackers and Δ*yejO* or Δ*fimE* (with and without 5% of D-mannose) target cells. Target survival after killing assays is presented in log10 CFUs. One-way ANOVA test was used followed by Tukey post hoc test to determine statistical significance using R (ns = nonsignificant, **P ≤ 0.005, ***P ≤ 0.0005, ****P ≤ 0.0001). Data represent three independent experiments.

To confirm that the formation of microcolonies observed was mediated by type 1 fimbriae, we next observed the formation of microcolonies in the *fimE* mutant with (5%) and without D-mannose (Ctrl) (Fig. 4A). D-mannose binds the FimH adhesin at the tip of type 1 fimbriae inhibiting adhesion (54) and agglutination to yeast (55). Indeed, we observed that adding D-mannose to the *fimE* mutant bacterial cultures inhibited the formation of microcolonies (Fig. 4A). The Δ*fimE* strain supplemented with D-mannose led to the reduction of the size of particles in comparison to the Δ*fimE* mutant without D-mannose (Fig. 4C). The analysis of particle sizes across multiple experiments further showed that the Δ*fimE* strain had a significantly greater average particle compared to the Δ*fimE* mutant supplemented with D-mannose (Fig. 4D).

In addition, type 1 fimbriated cells are known to promote yeast agglutination (55). Overnight cultures of various bacterial strains were mixed with rehydrated *Saccharomyces cerevisiae*, spotted on a glass slide, and observed macroscopically. As expected, *E. coli* Δ*yejO* did not agglutinate *S. cerevisiae* cells (Fig. 4B) in contrast to the solution containing the *fimE* mutant where agglutination was visible. The agglutination phenotype observed with the *fimE* mutant was inhibited by the addition of D-mannose, as expected.

Finally, we tested whether functional (i.e., adhesive) type 1 fimbriae were needed to promote resistance to T6SS-mediated killing. Therefore, we performed the competition assay in the presence or absence of D-mannose (Fig. 4E). As observed previously, the *fimE* mutant was protected against T6SS attacks. The addition of D-mannose to Δ*fimE* cultures to prevent the formation of microcolonies led to the loss of T6SS resistance to the same extent as the Δ*yejO* strain (Fig. 4E). In these conditions, growth of *C. malonaticus* and T6SS activity are not affected by the addition of 5% of D-mannose (Fig. S2A and B). We also performed the same experiments using a double *fimE fimH* deletion mutant and observed similar results (Fig. S3). Collectively, these findings highlight the implication of FimH adhesive interactions in the formation of microcolonies and resistance to T6SS-mediated killing. In addition, the interbacterial killing assays suggest that T6SS resistance exhibited by the *fimE* mutant is not due to single-cell spatial segregation driven by the fimbriae but more likely attributed to microcolonies formed through FimH.

### Formation of microcolonies by type 1 fimbriated target cells leads to their resistance to the T6SS

**Fig 5:**
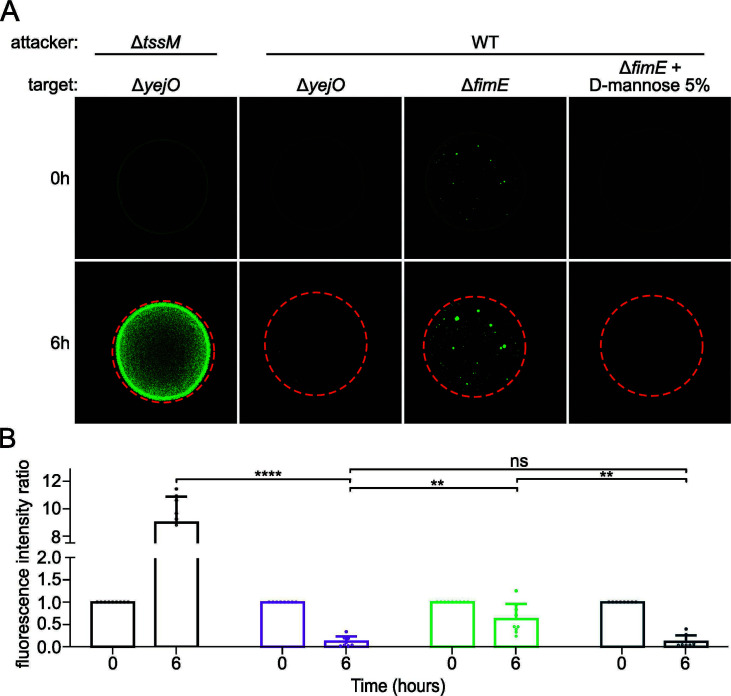
(A) Whole-colony microscopy reveals that type 1 fimbriae-dependent microcolonies confer resistance to the Δ*fimE* mutant against T6SS-mediated killing. Interbacterial killing was assessed using confocal microscopy and GFP-positive target cells. Attackers and targets grown overnight were mixed with a ratio of 1:1, spotted onto LB 0.8% noble agar in 12-well plates, and incubated at 37°C. The co-cultures were imaged every hour for 6 h. The colonies were delimited using orange dashed circles. (**B**) The killing rate was evaluated by assessing the total fluorescence inside the spot at 0 and 6 h using ImageJ and compared with the initial fluorescence rate (at 0 h). One-way ANOVA test was performed followed by Games-Howell post hoc test with Welch’s correction to determine statistical significance using R (ns = nonsignificant, **P ≤ 0.005, ****P ≤ 0.0001). Average ± s.d. of *n* = 6–9 replicates.

We have shown that D-mannose inhibited microcolony formation and suppressed the resistance to T6SS-mediated attacks. However, about 50% of the Δ*fimE* cells expressed type 1 fimbriae. In addition, the *fimE* deletion does not grant a complete resistance to T6SS attacks. Thus, we hypothesized that target cells inside microcolonies are protected, while individual cells are killed by the attacker. To test our hypothesis, we performed a time course interbacterial competition assay adapted from Granato et al. (29). Co-cultures were spotted directly onto LB-agar in 12-well plates and imaged every hour for 6 h to observe the GFP-positive target cells (Fig. 5A). The susceptibility of GFP-negative and GFP-positive target cells to T6SS killing was similar (Fig. S4). The total fluorescence inside the spot was then evaluated at 0 and 6 h (Fig. 5B), where the fluorescence intensity represents a measurement of target cells survival. When grown with a T6SS-deficient attacker, the Δ*yejO* target strain multiplied strongly as expected, reaching a 10-fold higher fluorescence intensity ratio at 6 h compared with *T*_0_. On the other hand, the Δ*yejO* target strain was eliminated after 6 h of co-culture with the T6SS-positive attacker. We observed the same result for the Δ*fimE* target supplemented with 5% of D-mannose, validating our previous assertions that the fimbrial structure by itself is not sufficient to confer resistance to T6SS. In condition without D-mannose supplementation, the Δ*fimE* mutant was found within microcolonies as well as single cells (i.e., weak fluorescence signal across the entire spot) at *T*_0_. Here, however, cells found in microcolonies survived the T6SS attack, while the fluorescence signal from the single cells was not detectable at 6 h. Overall, our results suggest that cells inside type 1 fimbriae-mediated microcolonies are protected from T6SS attacks, while individual cells are not.

## DISCUSSION

The discovery, 20 years ago, of the type 6 secretion system, a microbial weapon resembling the contractile tail of bacteriophages, changed our appreciation of bacterial dynamics within complex communities. Most studies on T6SS focus on various aspects of its biology, including biogenesis, assembly, function, and effector characterization. T6SSs inject toxic effectors directly into bacterial target cells. T6SS producers avoid autotoxicity by expressing effector-specific immunity proteins. There has been a growing interest in immunity-independent target cell defense mechanisms after the observations that envelope stress responses (26) and EPS production (29) could promote resistance to T6SS. This last study focused on the concept of spatial separation, which occurs through the formation of microcolonies, as a strategic means to evade T6SS attacks. For example, type 4 pili, which are known to drive the formation of microcolonies (56) in *Neisseria* species, were shown to promote survival when expressed in target cells through segregation of attacker and target cells (56).

In this work, we developed a high-throughput interbacterial competition assay that allowed us to rapidly identify mutants that were resistant to T6SS-mediated killing of *C. malonaticus* 3267. *C. malonaticus* is an interesting model organism to study T6SS biology as its T6SS activity is highly effective under typical laboratory conditions (Fig. 1). The majority of gene deletions identified in our HTIC assay that conferred resistance to T6SS attacks were primarily associated with transport and metabolism (degradation and energy production), but some of our hits were previously associated with cell motility and biofilm formation, including Δ*ypdB* (57) and Δ*ymgC* (58). The product of these genes would thus impact how attacker and target cells interact together. This suggests that the modulation of the interaction dynamics between attacker and target cells could be a main strategy by which target cells can evade T6SS attacks (28, 29, 59, 60). Another hit that exhibited resistance to T6SS activity is the *ldtB* mutant. LdtB functions as a periplasmic L,D-transpeptidase which cleaves the peptide bond between *meso*-diaminopimeloyl (*m*DAP) and D-alanine in the peptidoglycan (61, 62). The T6SS-dependent activity of *C. malonaticus* is associated with the amidase effector Tae4, which is present in the T6SS cluster. The Tae4 effector is a L,D-transpeptidase that induces cell lysis by cleaving the d-Glu-*m*DAP bond in gram-negative bacteria (21). A mutant with a slightly compromised cell wall, in which the effector might potentially act, could unexpectedly exhibit resistance. Further research is required to investigate and test this concept.

We focused our analysis on the *fimE* mutant, a regulator of type 1 fimbriae production, which interestingly, appeared to confer partial protection against T6SS. Notably, the expression of type 1 fimbriae is governed by a phase variation mechanism, resulting in a heterogeneous phenotype within a clonal population. This variability could account for the observed resistance conferred by the *fimE* mutant in only two out of the three replicates. Consequently, it underscored the necessity for further investigations.

Type 1 fimbriae are rod-shaped structures found at the surface of numerous bacterial species including enteric pathogen such as enteropathogenic and enterohemorrhagic *E. coli* (63, 64). Some studies have highlighted a link between type 1 fimbriae expression and T6SS in attacker cells (65). For instance, in avian pathogenic *Escherichia coli* strains, mutations of T6SS genes decrease the expression of type 1 fimbriae, leading to lower adhesion and epithelial cells invasion (66). Our findings demonstrate that type 1 fimbriae also play a defensive role in target cells.

Our initial hypothesis was that the *fimE* mutant overexpressed type 1 fimbriae genes, resulting in their increased production. Electron microscopy and agglutination assays confirmed that the *fimE* mutant indeed increased the production of type 1 fimbriae compared to wild type (Fig. 3). The FimH adhesin, located at the tip of the type 1 fimbriae and responsible for adherence to mannose-specific receptors on host cell surfaces (67), has been previously associated with stress response behaviors, such as the internalization of fimbriated *E. coli* by macrophages in response to lethal concentrations of antibiotics (68). Moreover, adhesion appears to be an important mechanism for protection against environmental stress such as shear stress (52, 69). Adhesion mediated by type 1 fimbriae is the first step in biofilm formation (70, 71), where adherent bacteria organize themselves into microcolonies embedded within a self-produced matrix of exopolysaccharide, extracellular DNA, and proteins (72). Indeed, our whole-colony microscopy assay demonstrated that type 1 fimbriae enabled the establishment of microcolonies (Fig. 3) and that target cells within these microcolonies were protected from T6SS-mediated killing in contrast to individual bacterial cells which were efficiently killed (Fig. 5). Moreover, whole-colony microscopy of Δ*fimE* mutant in the presence of D-mannose showed that isolated fimbriated cells were killed, leading us to conclude that fimbriae alone are not sufficient to protect against T6SS attack, and emphasized that the formation of microcolonies is providing this resistance.

The synthesis of fimbriae or other adhesins such as autotransporters (73, 74), promoting the formation of microcolonies (73, 74), could represent a widespread and nonspecific defense mechanism against T6SS attacks from diverse bacteria. Accordingly, a *fim* locked-OFF strain of uropathogenic *E. coli* CFT073 still demonstrated resistance to T6SS attacks, albeit to a lesser extent than a locked-ON strain (data not shown). *E. coli* strain CFT073 possesses multiple fimbriae operons that are expressed in a coordinated manner (75). This raises questions about whether other types of fimbriae could also promote the resistance phenotype against T6SS attacks. Additionally, such structures may confer protection against various adverse stresses, including antimicrobials and reactive oxygen species. For instance, Schembri et al. demonstrated that bacterial aggregation induced by the adhesin Ag43 expression enhances survival in the presence of hydrogen peroxide (76), suggesting that the formation of microcolonies may offer broad protection against multiple stresses.

Importantly, the T6SS is not the sole contact-dependent weapon found in gram-negative bacteria. Certain contact-dependent growth inhibitions (CDI) are known to be secreted by T4SS (77) and T5SS (78). Microcolonies formed by type 1 fimbriae may therefore also provide protection to prey cells against attacker cells that use these other CDI systems. Further research is necessary to fully understand the role of type 1 fimbriae in the protection of cells under diverse stress conditions.

One of our hypotheses, supported by electron microscopy findings indicating that 50% of cells are fimbriated in the Δ*fimE* mutant, is that isolated cells constitute the nonfimbriated cell fraction of the Δ*fimE* mutant and that microcolonies were formed by the fimbriated cells. It is also possible, however, that nonfimbriated cells are also part of the microcolonies. It would therefore be interesting to identify the fimbriae-producing cells within the population in order to observe if cheaters can profit from this collective behavior. Multicellular lifestyles are prone to the presence of cheaters in the population (79). Because type 1 fimbriae are regulated through phase variation, this represents an intriguing possibility and could represent a selfless defense mechanism against the T6SS assault.

T6SS plays a crucial role in shaping microbial populations in various environments, including the gastrointestinal microbiota (34, 35, 80). Indeed, T6SS are prevalent in enteric bacteria (81). Type 1 fimbriae represent a prevalent adhesive organelle found in various members of the Enterobacteriaceae family and play a significant role as virulence factors (82). In some bacteria, those determinants act simultaneously to adhere and invade host cell (66). Studying the relationship between T6SS and type 1 fimbriae expression and, more broadly, adhesin structures in pathogenic versus host-associated bacteria *in vivo* would be an interesting line of research to elucidate the networks of genetic interactions involved in competition between bacteria.

## MATERIALS AND METHODS

### Strains, media, and chemicals

Bacterial strains and plasmids used in this study are listed in Table 1. Bacterial cultures were grown in Luria broth-Lennox medium (10 g/L NaCl, 5 g/L yeast extract, 5 g/L tryptone, and 15 g/L agar when needed). When required, the media were supplemented with kanamycin (50 µg/mL), ampicillin (100 µg/mL), diaminopimelic acid (0.3 mM), spectinomycin (120 µg/mL), chloramphenicol (37 µg/mL), and D-mannose 5%. Liquid cultures of *C. malonaticus* 3267 and *E. coli* BW25113 were both grown at 37°C with agitation (180 and 80 rpm, respectively).

**TABLE 1:** Bacterial strains and plasmids used in this study

Strain or plasmid	Description	Source
*Cronobacter malonaticus* 3267	Wild type	This study
Δ*tssM::aadA(smR*)	*tssM* gene deletion mutant from *Cronobacter malonaticus* 3267	This study
Keio collection	Mutant collection of all nonessential genes from *E. coli* K-12 BW25113. F−, Δ(*araD-araB*)567, λ−, rph-1, Δ(*rhaD-rhaB*)568, *hsdR*514	KEIO collection (40)
Δ*yejO::nptII(kanR*)	*yejO* pseudo gene deletion mutant from *E. coli* K-12 BW25113. Considered as *WT*	This study
Δ*fimE::nptII(kanR*)	*fimE* gene deletion mutant from *E. coli* K-12 BW25113	This study
pCA24N	Cm^R^, IPTG-inducible promotor	ASKA collection (43)
pCA24N-*fimE*	Cm^R^, IPTG-inducible promotor, expression of FimE	ASKA collection (43)
pKD3	Template plasmids for frt-flanked cat cassette. cat cassette originally came from pSC140	(83)
pSIM19	Sm^R^, pSC101 *repA^ts^*	(84)
pSIM6	Amp^R^, pSC101 *repA^ts^*	(85)
pTrcGFP	Amp^R^, green fluorescent protein expression, pTrc99a backbone	This study

### Strain construction

Gene deletion in *C. malonaticus* 3267 was done using the lambda red recombinase system protocol (83) with minor modifications. Briefly, a spectinomycin resistance cassette was amplified from plasmid pSIM19 using primers (listed in Table 2) carrying at the 5′ end 30-nucleotide extensions homologous to regions adjacent to the gene to be deleted. PCR products were purified, then 2 µL was transformed into *C. malonaticus* 3267 electro-competent cells carrying the red recombinase pSIM6 vector. Transformants were selected on spectinomycin plates and verified by colony PCR using primers listed in Table 2.

**TABLE 2:** Primers used in this study

Primer type and gene	F/R	Sequence
Gene deletion		
*tssM*	F	taaagcgctgcaaaccaccgccgggcgcgatctcttcagcgtgtaggctggagctgcttc
*tssM*	R	gctaatcgcagggtaatgactcattatccgttccttgcggcatatgaatatcctccttag
Colony PCR verification		
*tssM*	F1	acgtgattttccaggaagcgg
*tssM*	F2	ccacggaatgatgtcgtcg
*tssM*	R1	ggagtgaataccacgacgat
*tssM*	R1	cgacgacatcattccgtgg

### Growth curves

Growth was assessed using overnight culture and adjusted to an optical density (OD_600_) of 1, diluted 1/100, and transferred to 96-well plates. The strains were grown at 37°C in a Spark 20M multimode microplate reader (TECAN), where the OD_600_ was measured every 20 min for each strain.

### Particles analysis using confocal microscopy

Δ*yejO* and Δ*fimE* prey strains were cultured in liquid overnight and were next adjusted to an OD_600_ of 1. Then, 5% D-mannose was added to Δ*fimE* prey culture when required, and 2 µL was spotted onto LB 0.8% noble agar in 12-well plates in duplicate. The particles were observed using a 20× magnification FV3000 Olympus confocal microscope for three independent experiments. For each experiment, strains were spotted in duplicate. Three images were taken at random coordinates for each duplicate. Particle sizes were assessed using the ImageJ “Analyze Particles” function.

### Interbacterial competition assay

*C. malonaticus* 3267 WT and Δ*tssM* mutants were used as attacker cells. *E. coli* BW25113 Δ*fimE* and Δ*yejO* were used as target cells for interbacterial competition assay. Attackers and targets were grown in liquid media overnight. The cultures were adjusted to an OD_600_ of 1. And, 5% D-mannose was added to target cultures when required. Approximately 10^7^ bacteria were combined at a ratio of 1:1, then 5 µL was spotted onto sterile nitrocellulose filters on LB agar plate and incubated for 24 h at 37°C. The co-cultures were resuspended in 1 mL of LB broth. Serial dilutions were plated onto LB selective medium to assess the number of CFUs of surviving target cells. The experiment was repeated three times independently. For the complementation assay, *E. coli* BW25113 Δ*yejO* pCA24N, Δ*fimE* pCA24N, and Δ*fimE* pCA24N-*fimE* were used as targets, and the expression of genes from pCA24N vector was induced with 10 µM of isopropyl-β-D-thio-galactopyranoside in overnight liquid cultures and competition agar plates used for interbacterial competition.

### High-throughput interbacterial competition assay

Attackers *C. malonaticus* 3267 WT and Δ*tssM* mutants were grown in overnight liquid cultures at 37°C. Next day, the cultures were pinned on agar plates with 1,536-pin pad using the Rotor HDA (Singer Instruments, United Kingdom), then incubated 1 h at 37°C. The Keio collection was grown, in parallel, for 2 h in 384-well plates at 37°C, directly from 384-well plates stored at −80°C. Keio mutants were then pinned onto competition agar plates and incubated for 24 h at 37°C. Survival was assessed by pinning onto kanamycin selection plates. Surviving was assessed for each replicate using binary value; any growth refers to resistance (=1), no growth means that the mutant is sensitive (=0). In this experiment, a mutant was considered resistant if there was growth in at least two of the three replicates.

### Yeast agglutination assay

Strains used in this assay were grown overnight in liquid media. The cultures were adjusted to an OD_600_ of 6 and then centrifuged at 5,000 rpm for 5 min and then resuspended in 5 mL of phosphate buffered saline (PBS, pH 7.2). Yeast cells were resuspended in 5 mL of PBS. And, 5% D-mannose was added when required. Cells were mixed at a ratio of 1:1, then 20 µL was spotted onto glass slides.

### Electron microscopy

Twenty-four-hour colonies grown on agar plates at 37°C for 24 h were resuspended in HEPES buffer and then deposited on carbon-supported grids at room temperature. Samples were negatively stained with 2% of uranyl acetate. Negatively stained bacterial cells were examined with a TECNAI G2 electron microscope. Fifty pictures were acquired for each condition. All bacteria were considered for counting the number of fimbriae per cell.

### Interbacterial competition assay in macrocolony and observation using confocal microscopy

Interbacterial competition assays were performed as described by Granato et al. (29) with minor modifications. Briefly, target cells carrying the pTrcGFP strong gfp-expressing vector were grown in liquid media overnight with 100 µg/mL of ampicillin. The cultures were adjusted to an OD_600_ of 1. D-mannose (5%) was added when required. Approximately 10^6^ bacteria were combined at a ratio of 1:1, then 2 µL was spotted on LB 0.8% noble agar in 12-well plates and incubated at 37°C, and images were acquired every hour for 6 h using a 20× magnification FV3000 Olympus confocal microscope. The killing rate was evaluated by assessing the total fluorescence inside the spot at 0 and 6 h using ImageJ.

### Statistical analysis

All experiments were carried out at least three times with at least two biological replicates for each experiment except for the electron microscopy assay. Statistical analyses were made using R. The normal distribution of the data was first assessed, followed by a Levene’s test to examine the homogeneity of standard deviations across samples. The choice of whether to use parametric or nonparametric tests to analyze the data was made in accordance with the results obtained for the normality tests. The choice between ANOVA and two-group comparison tests depended on the number of groups under analysis.

## SUPPLEMENTAL MATERIAL

Supplemental material mbio.02553-23-s0001.docx Supplemental figures and tables.

ASM does not own the copyrights to Supplemental Material that may be linked to, or accessed through, an article. The authors have granted ASM a non-exclusive, world-wide license to publish the Supplemental Material files. Please contact the corresponding author directly for reuse.
