# Supplementary material for: Type 1 fimbriae-mediated collective protection against type 6 secretion system attacks
Source: mBio. 2024 Mar 18;15(4):e02553-23. doi: 10.1128/mbio.02553-23 (PMC11005336; doi:10.1128/mbio.02553-23)

**Supplemental Materials**

**Type 1 fimbriae-mediated collective protection against type 6 secretion system attacks**

Margot Marie Dessartine^1^, Artemis Kosta^3^, Thierry Doan^2^, Éric Cascales^2^, Jean-Philippe Côté^1#^

^1^ Département de biologie, Faculté des sciences, Université de Sherbrooke, Sherbrooke, QC J1K 2R1, Canada.

^2^ ​Laboratoire d'Ingénierie des Systèmes Macromoléculaires (LISM, UMR7255), Institut de Microbiologie de la Méditerranée, Aix Marseille Univ, CNRS, Marseille, France

^3^ Plateforme de microscopie, Institut de Microbiologie de la Méditerranée (IMM, FR3479), Aix-Marseille Univ, CNRS, Marseille, France

# Correspondence may be addressed to:

Jean-Philippe Côté

Tel :1-819-821-8000 ext. 65280

Email: jp.cote@usherbrooke.ca

**This Supplemental Material includes:**

Supplementary Figures 1 to 4

Supplementary Tables 1 and 2


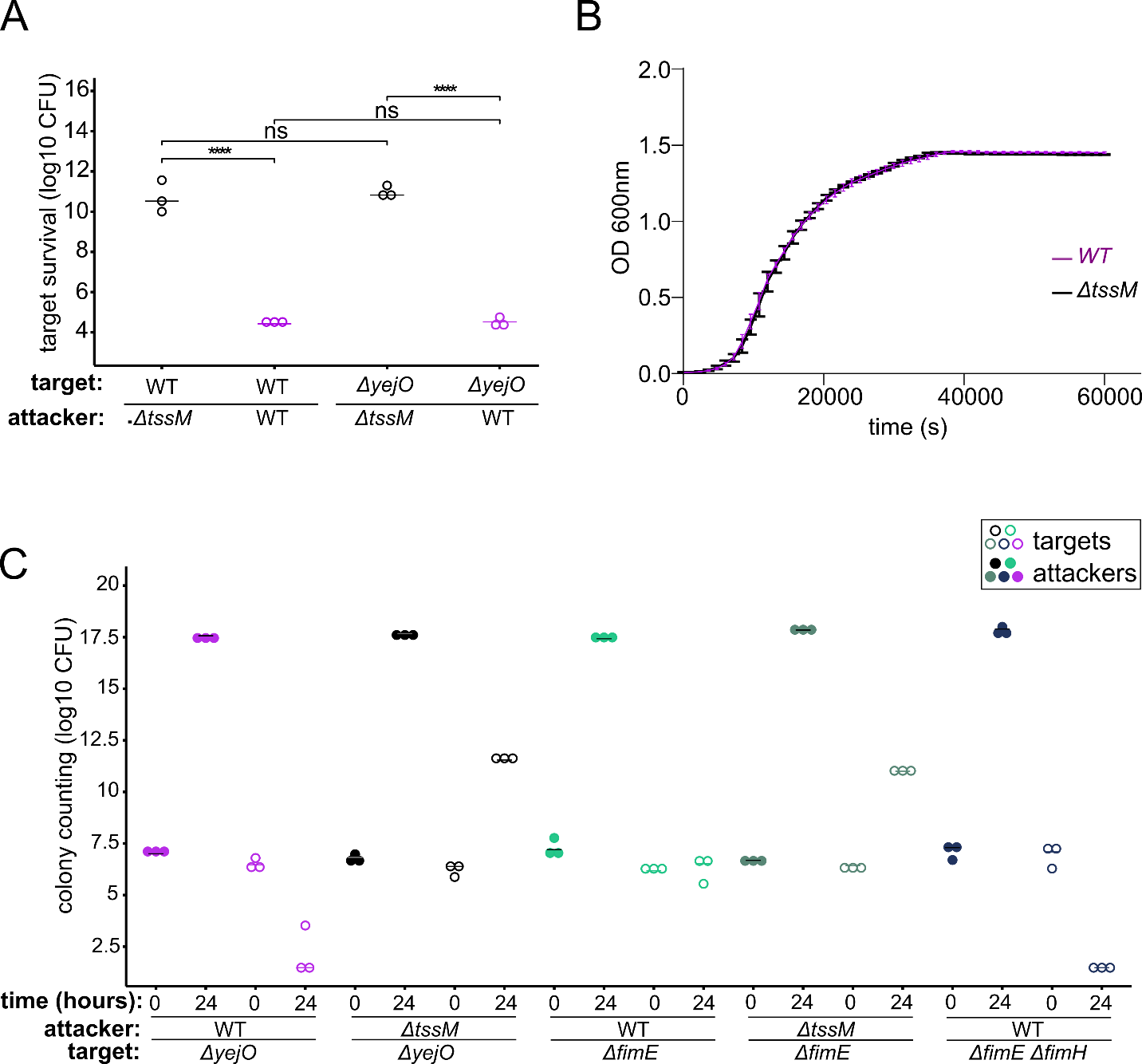


**Supplementary Fig. 1:** (A) Interbacterial competition assay between C. malonaticus 3267 WT or ΔtssM attackers and ΔyejO and BW25113 WT target cells. Target survival is presented in Log10 CFU. One-way Anova test was used followed by Tukey post-hoc test to determine statistical significance (ns=nonsignificant, ****p≤0.0001). Data represent three independent experiments (B) Growth curves of C. malonaticus 3267 WT and ΔtssM is shown. Overnight cultures of attacker strains were diluted 100 times, transferred to 96-well plates, and grown for 17 hours. OD_600_ was measured every 20 min for each strain. The data represent the mean (±SD) of three independent experiments. (C) Interbacterial competition assay between WT or ΔtssM attackers and E. coli ΔyejO, ΔfimE and ΔfimEH target cells. Attacker and target survival is assessed at 0h and 24h after an interbacterial competition assay and is presented in Log10 CFU. Filled circles represent attackers and empty circles represent targets.


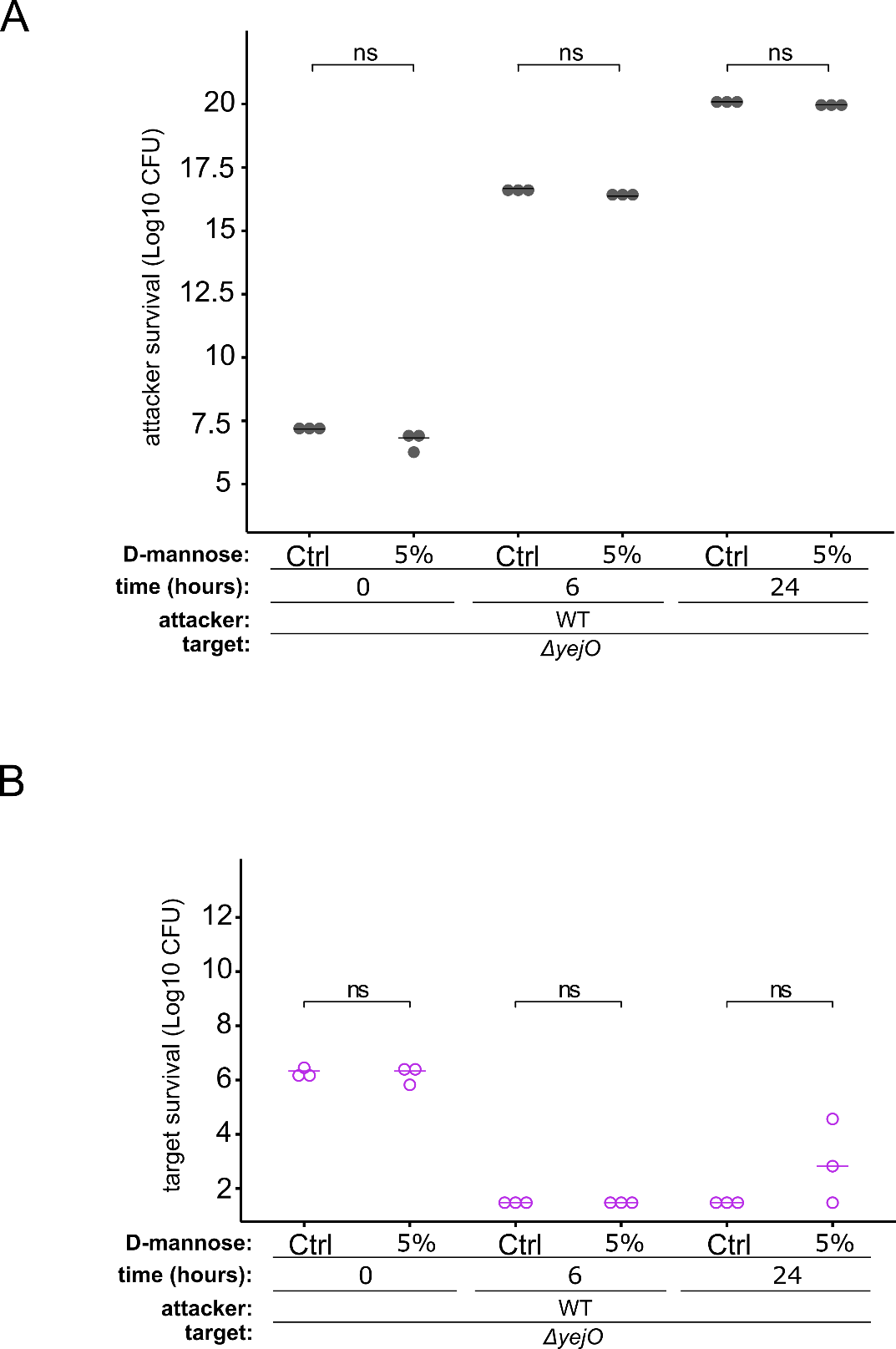


**Supplementary Fig. 2:** Interbacterial competition assay between C. malonaticus *3267* WT attacker and Δ*yejO* target cells (with and without 5% of D-mannose). (A) Attacker survival at 0h, 6h and 24h following interbacterial killing assay is presented in Log10 CFU. (B) Target survival at 0h, 6h and 24h following interbacterial killing assay is presented in Log10 CFU. One-way Anova test was used followed by Tukey post-hoc test to determine statistical significance using R (ns=nonsignificant). Data represent three independent experiments.


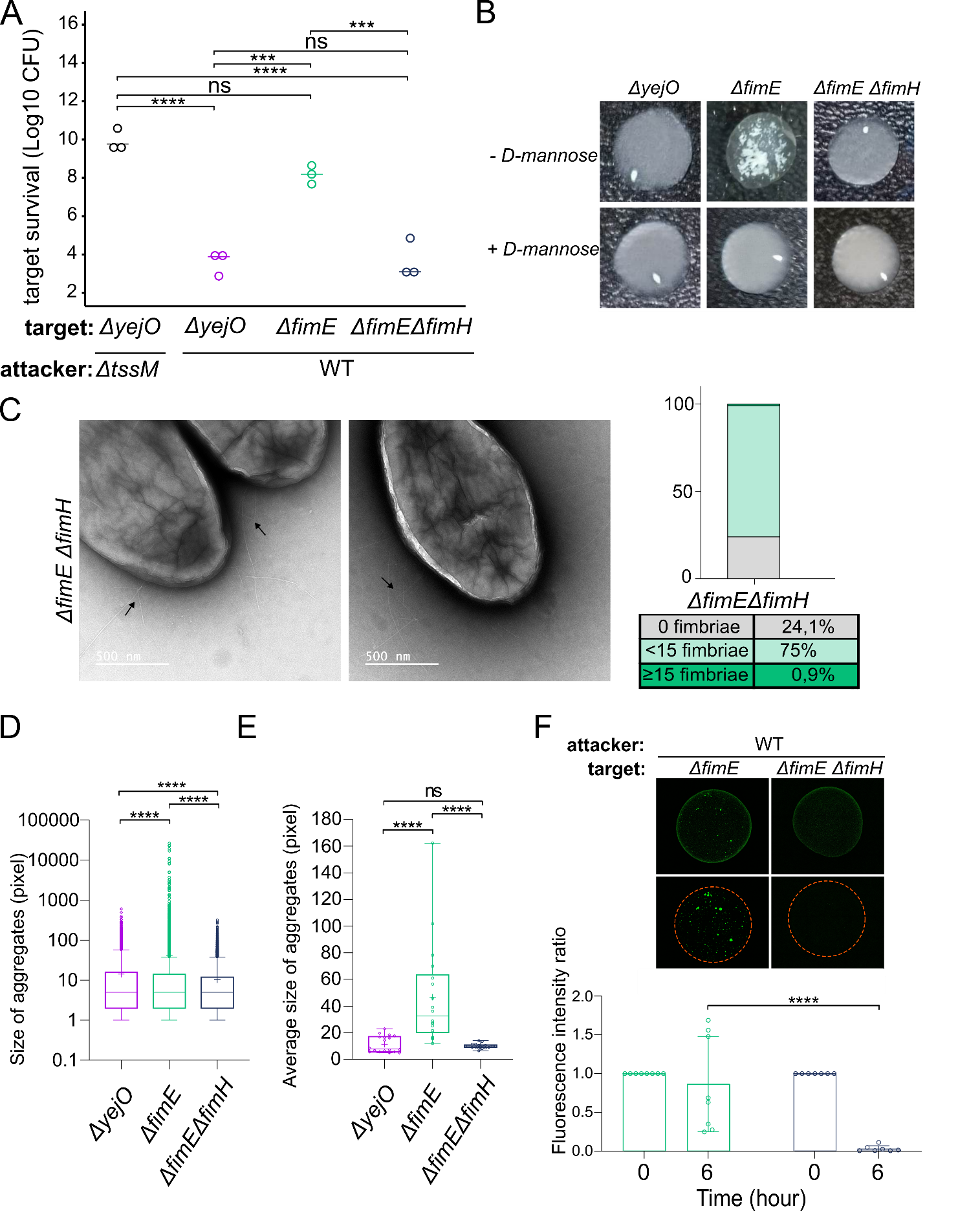
**Supplementary Figure 3:** (A) Interbacterial competition assay between *C. malonaticus 3267* WT or Δ*tssM* attacker and Δ*yejO*, Δ*fimE* and Δ*fimEfimH* target cells. Target survival is presented in Log10 CFU. One-way Anova test was used followed by Tukey post-hoc test to determine statistical significance (ns=nonsignificant, ***p≤0.0005, ****p≤0.0001). Data represent three independent experiments. (B) Yeast agglutination assay with and without D-mannose as a competitive ligand to inhibit adhesion. A positive agglutination phenotype is observed for the Δ*fimE* mutant. A negative agglutination phenotype is characterized by a cloudy appearance of the mixture and observed for the Δ*fimEfimH* double deletion mutant. (C) Electron microscopy of fimbriated cells. Negatively stained preparation of a 24h stationary culture of *E. coli* BW25113 Δ*fimEfimH*, grown on LB agar plate. (D) Box plot of overall particles size of Δ*fimEfimH* mutant versus Δ*yejO* and Δ*fimE* mutants from fluorescence microscopy images. Box plots extend from the 5th to 95th percentiles. Cross: mean, crossing line: median. A nonparametric Kruskal Wallis test was performed followed by Dunn’s post-hoc test to determine statistical significance (****p≤0.0001). (E) Box plots of the average size particles of each random point images. Box plots extend from min to max, showing all points. Cross: mean, crossing line: median. One-way Anova test was performed followed by Games-Howell post-hoc test with Welch’s correction to determine statistical significance (ns=nonsignificant, ****p≤0.0001). (F) Confocal microscopy imaging of interbacterial killing using the same method as in Fig. 5. The killing rate was evaluated by assessing the total fluorescence inside the spot at 0h and 6h using ImageJ and compared with the initial fluorescence rate (at 0h). Unpaired t-test was performed to determine statistical significance using R (****p≤0.0001). Average ± s.d of n = 7-8 replicates.


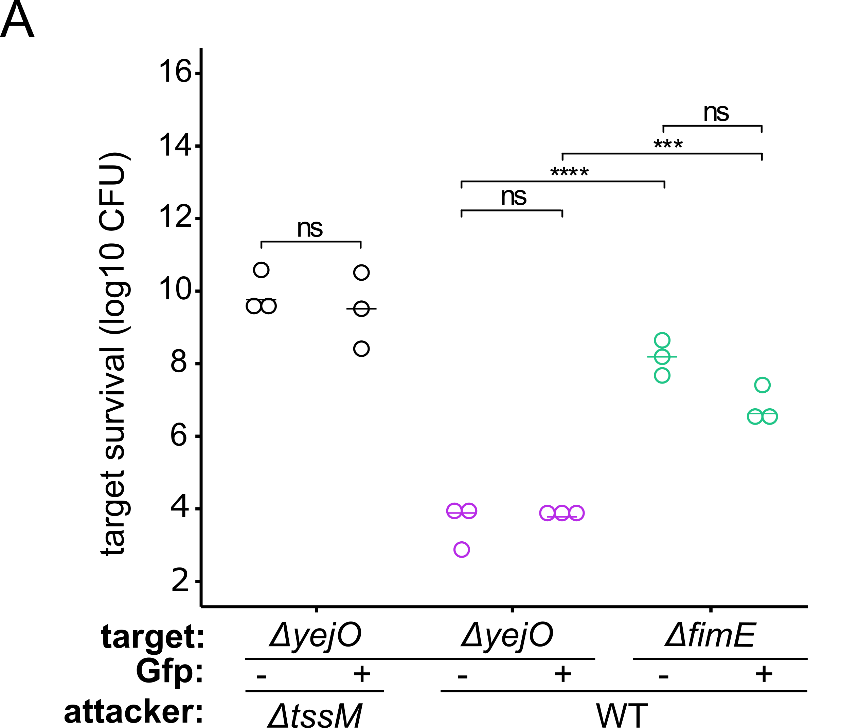


**Supplementary Fig. 4**: Interbacterial competition assay between *C. malonaticus 3267* WT or Δ*tssM* attacker and Δ*yejO* and Δ*fimE* target cells or Δ*yejO* GFP+ and Δ*fimE* GFP+ target cells. Target survival following interbacterial killing assay is presented in Log10 CFU. One-way Anova test was used followed by Tukey post-hoc test to determine statistical significance (ns=nonsignificant, ***p≤0.0005, ****p≤0.0001). Data represent three independent experiments.

**Supplementary table 1:**


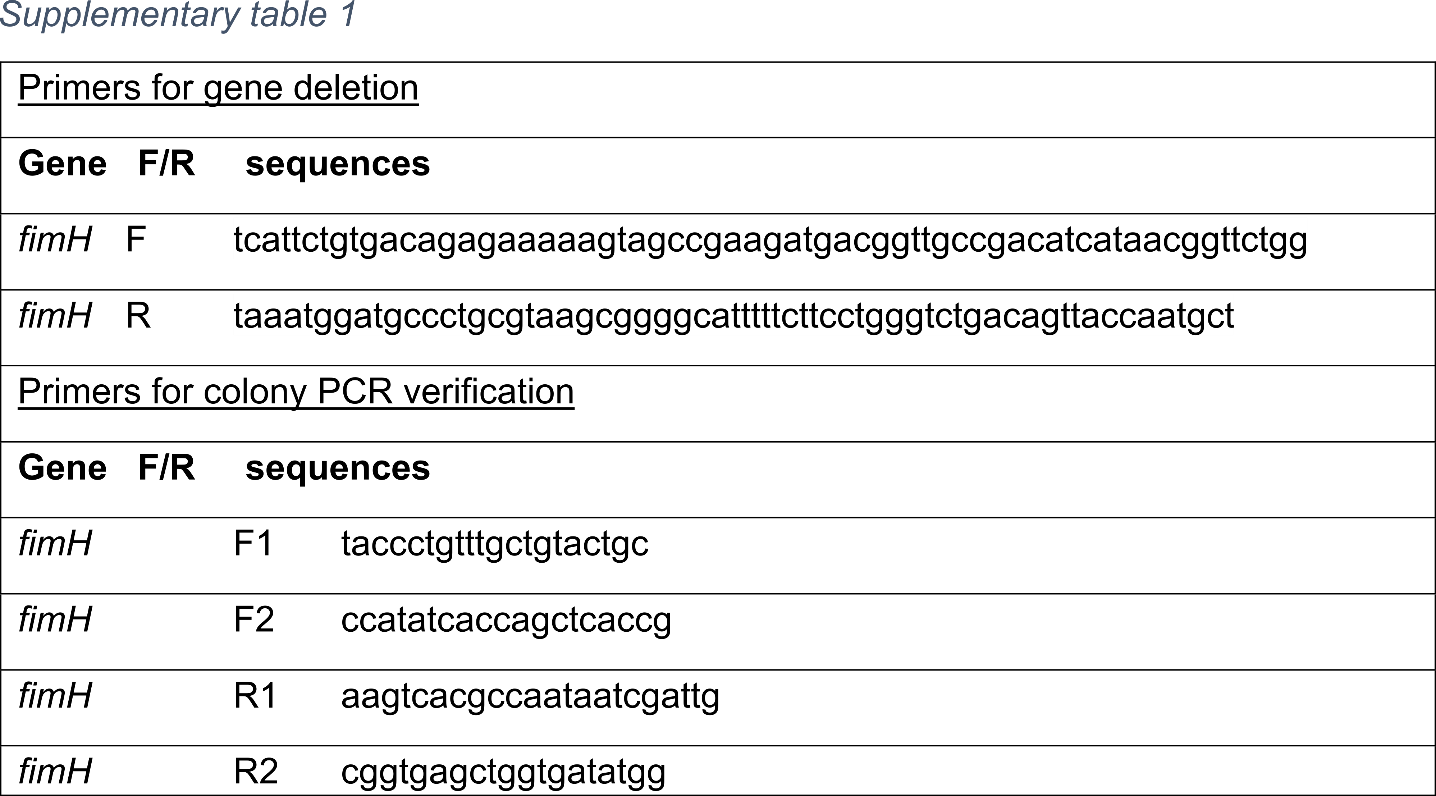


**Supplementary table 2:** summary of hits obtained following the HTIC assay.


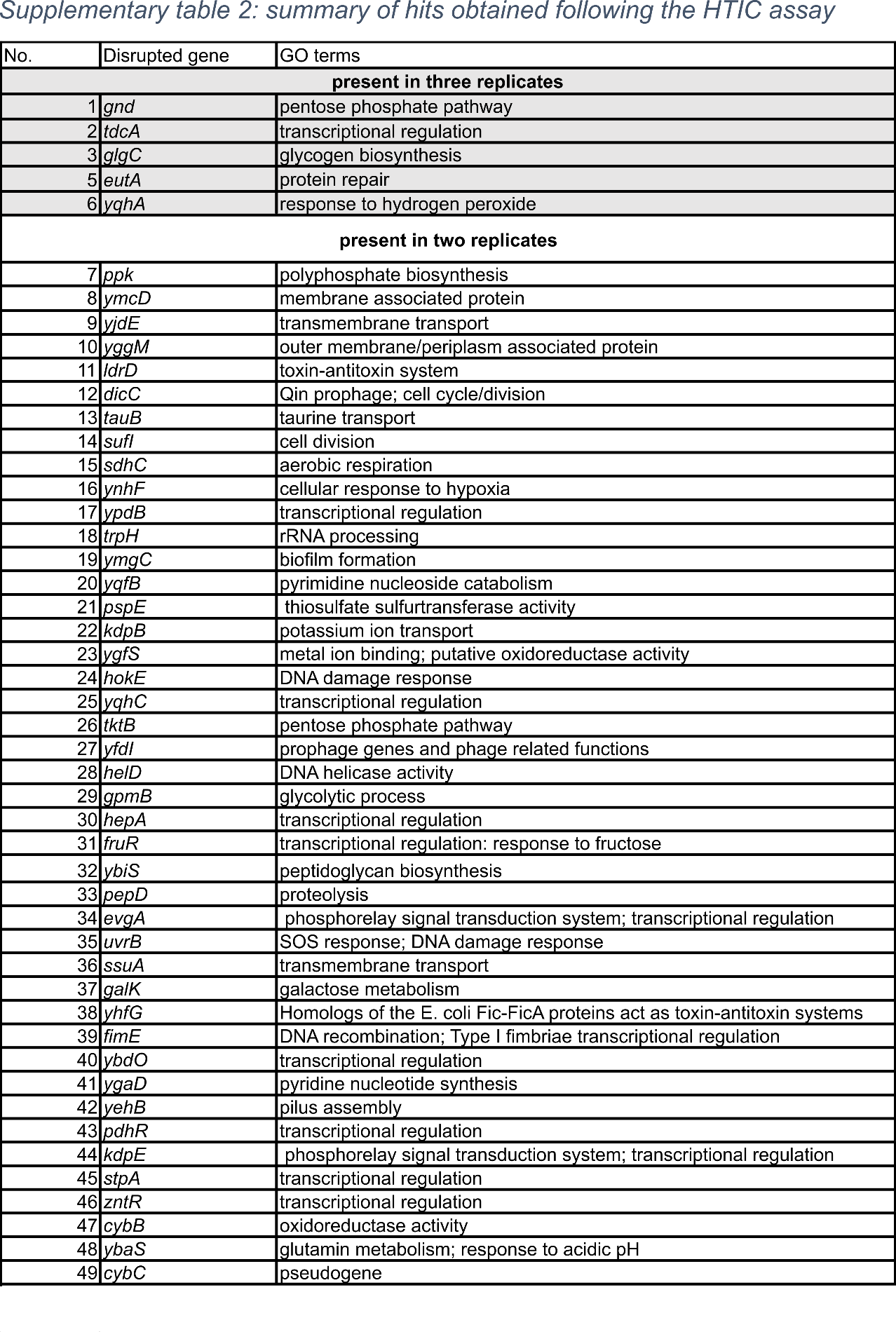

Supplement: Supplemental material — Supplemental figures and tables. [file mbio.02553-23-s0001.docx]
